# Modern Sociology

**Published:** 1902-01-18

**Authors:** 


### MODERN SOCIOLOGY.

### WOMEN'S RESTAURANTS.

### II.—SOLUTIONS—SO FAR.

WE have recently visited several of the women's restaurants in London, which cater for the working-class woman, and in all the same answer to the question, "Does it pay?" has been received: "We cover all expenses except rent." And for this large item resort is had to subscriptions. So far, then, these restaurants have not solved the problem; but it is interesting to see what they have done. The "Welcome" and the "Churchill" are the largest we have seen; each caters for some 400 or 500 girls and women daily.

"They are established for providing the girls with wholesome food at reasonable prices," says one of the annual reports. "How much this is needed by girls of this class may be judged by the fact that some of these City girls (fair specimens of their class) were refused admission to a Benefit Society on the ground that they were so sadly below par; they were not sufficiently fed. What they wanted, the doctor said, was not medicine but food. At the 'Welcome' hundreds of girls come for soup and bread at 1½d. and meat puddings at 2d., which could not be surpassed at the price at any eating house in London; and, besides this, free dinner tickets are sometimes given to girls who are found on the borders of starvation, having no work and consequently no food. These tickets tide them over the times of distress and difficulty until they can resume their work." The restaurant is an adjunct to other work, social and religious, and as no effort appears to be made to "make it pay" in any commercial sense, it is perhaps hardly fair to put it to a severe test. The class of girls provided for may be seen from a further extract from the report:—

"Few people probably realise that in London alone over 150,000 women and girls are employed in factories and workrooms. They are machinists, needlewomen, feather curlers, bookbinders, and makers of cardboard boxes, sweets, envelopes, umbrellas, ties, corsets, shirts and collars, button-holes, and hats and bonnets. It has indeed been said that there is scarcely an article of apparel worn by men or women which, in some way or another, does not pass through the hands of these girls; besides many other things which are in constant use among us."

The "Southwark" is a women's restaurant, under the management of Sister Mary Gabriel, of the Working Girls' Home in Nelson Square, Blackfriars. The meals are both cheap and good; but the paragraphs in the annual report are introduced with "The subscription list is still insufficient." In an interview with Sister Mary Gabriel we learnt that it was good management, clean tablecloths, and attention to the general details of cooking and serving the food that had rescued the restaurant from the condition into which, some seven years ago, under other management, it had sunk; and certainly we found, on going in for a dinner, that the meals were nicely prepared, and that a business-like air pervaded the establishment. The following is a specimen tariff:—

| | |
|---|---|
| A plate of English beef, mutton, or veal ... | 3d. |
| Fish, boiled or fried ... ... ... ... | 2d. and 3d. |
| Meat pie ... ... ... ... ... | 3d. |
| Stew or curry ... ... ... ... ... | 2d. and 3d. |
| Greens or potatoes ... ... ... ... | 1d. |
| Fruit or jam tart, or any kind of pudding ... | 1d. |
| Cup of tea ... ... ... ... ... | ½d. and 1d. |

This restaurant is a great boon to women and girls whose work lies on the Southwark side of the river, and we noticed that they ate a really substantial meal, the odds and ends (sardines, pickles, etc.) not being to the fore at all. It is evident that girls can be trained to require the more substantial food; and at the Working Girls' Home it has been found that, although at the beginning of their residence there girls are sometimes actually ill through having enough to eat, the habit grows upon them, and the sister in charge told us that the Sunday dinner-hour was a time of real enjoyment to her, for the girls so thoroughly enjoyed their food and appreciated the luxury of being able to sit over it for a full hour instead of having to hurry back to work. She has noticed a marked improvement in the factory girl in many ways during the last sixteen years, and good food and plenty of it is an essential feature of the "Home."

We have already alluded to the Maurice Hostel, Women's Branch. In the Shepherdess Walk is a small restaurant for the same class of girls as those catered for at the other restaurants mentioned. The premises, formerly belonging to a fryer of eels, can only accommodate about twenty-five girls; and here, again, subscriptions have to be drawn upon to pay rent. But Miss Eves would be very glad to make the restaurant self-supporting, and she detailed to us a scheme by which, admitting that men should also be catered for, a sound commercial business might be made, the profits on the men's dinners going to help the deficit on the women's. That the admission of men to any such scheme would have the effect of sending up the profits is evident; for at the "Southwark," where on one occasion the workmen doing some repairs on the premises were catered for, there was a marked difference in the week's takings. By running the restaurant in connection with a co-operative store, it would, it is thought, be quite possible to make a thoroughly sound investment out of a mixed restaurant and to pay a small dividend to shareholders. The girls might be given half or three-quarters the quantity of the man's portion, while the quality of each might be the same. There would be separate rooms for men and women; for it has been pointed out that, although the woman accepts less than the man for equal work, her pride shrinks from letting him see how little she is able to spend on her food.

The legal provision of a separate room from the workrooms, even if meals were provided here by the employers, does not meet the case. The law has fixed the hour for dinner in all factories and workshops under the Act; and if the mid-day meal were provided on the premises, it would make evasion of the law still more easy than it is now. Overtime in the dinner-hour is still, we are informed, too frequently worked "on the quiet"; and it is far better that the girls should be obliged to turn out of the building and get an hour's change of scene as well as of atmosphere. "They are already too much in the hands of the employers," said Miss Eves; "and they would be still more so if he were to provide the meals." Whether women could be induced to give a restaurant for them only, sufficient support to make it safe appears to be an open question; and we have not heard of anyone who has, so far, made a fortune in women's restaurants, though there is plenty of evidence that mixed restaurants pay a large profit (see Mr. Pearce's contribution to "Fortunes Made in Business.") Miss O'Kell, to whose letter we have before alluded, is sanguine about results. "I am convinced," she says, "that it would not be difficult to remedy this state of things by starting special restaurants where good hot meals, at cheap rates, could be obtained. Indeed, I am of opinion that it would be possible to raise capital for such a purpose; and if a sufficient number of these restaurants were started, and well managed, financial success might be counted upon and a small dividend paid to investors. Employers would doubtless liberally encourage such an undertaking, and in so doing would be acting in their own interests, while I am sure that many of the ladies who wear the beautiful gowns and millinery which these clever workers produce would gladly become shareholders, and so help to confer a boon on those who are labouring for them."

But it has been found in the experience of the "Southwark" that employers are too apt to consider that their responsibility with regard to their workpeople does not extend to meals; and we should prefer to see the scheme worked out on a larger and perfectly independent basis.

